# A Review of the Transition Region of Membrane Electrode Assembly of Proton Exchange Membrane Fuel Cells: Design, Degradation, and Mitigation

**DOI:** 10.3390/membranes12030306

**Published:** 2022-03-09

**Authors:** Daijun Yang, Yongle Tan, Bing Li, Pingwen Ming, Qiangfeng Xiao, Cunman Zhang

**Affiliations:** School of Automotive Studies & Clean Energy Automotive Engineering Center, Tongji University, 4800 Cao’an Road, Shanghai 201804, China; yangdaijun@tongji.edu.cn (D.Y.); tanyongle@tongji.edu.cn (Y.T.); pwming@vip.sina.com (P.M.); xiaoqf@tongji.edu.cn (Q.X.); zhangcunman@tongji.edu.cn (C.Z.)

**Keywords:** proton exchange membrane fuel cell, membrane electrode assembly, transition region, durability, degradation, mitigation, design

## Abstract

As the core component of a proton exchange fuel cell (PEMFC), a membrane electrode assembly (MEA) consists of function region (active area), structure region, and transition region. Situated between the function and structure regions, the transition region influences the reliability and durability of the MEA. The degradation of the electrolyte membrane in this region can be induced by mechanical stress and chemical aggression. Therefore, prudent design, reliable and robust structure of the transition region can greatly help avoid early failure of MEAs. This review begins with the summarization of current structural concepts of MEAs, focusing on the transition region structures. It can be seen that aiming at better repeatability and robustness, partly or total integration of the materials in the transition region is becoming a development trend. Next the degradation problem at the transition region is introduced, which can be attributed to the hygro-thermal environment, free radical aggression, air pressure shock, and seal material decomposition. Finally, the mitigation approaches for the deterioration at this region are summarized, with a principle of avoiding the exposure of the membrane at the edge of the catalyst-coated membrane (CCM). Besides, durability test methods of the transition region are included in this review, among which temperature and humidity cycling are frequently used.

## 1. Introduction

**Figure 1:**
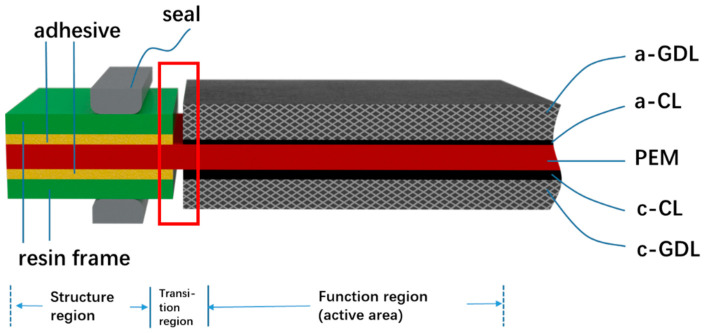
Schematic diagram of the primary structure of an MEA.

With increasing concerns over fossil fuel energy consumption and environmental issues, lithium ion batteries (LIB) [1], redox flow battery (RFB) [2], and proton exchange membrane fuel cells (PEMFCs) have attracted more and more attention for their zero carbon emission and availability. Among them, hydrogen PEMFCs have emerged as a leading candidate in the renewable energy industry due to its high efficiency, high power density, and rapid replenishment of energy. Recently, rapid progress in PEMFC technology has initiated the fuel cell vehicle (FCV) market. Nearly every major auto maker is developing FCVs, meanwhile more and more newbies are entering the arena. Emerging PEMFC applications almost cover all the types of transportation, including material handling forklift, passenger cars, light duty vehicles, medium duty delivery trucks, city buses, heavy duty trucks, trains and aircraft [3]. Many obstacles still stand in the way of the commercialization of PEMFC vehicles such as initial cost and insufficient durability. So far, researchers have conducted many innovative studies on fuel cell materials, components, stacks, and systems to promote the commercialization of PEMFCs [4]. A PEMFC stack has a determined number of unit cells, associated in series, along with supporting components to produce electricity of the desired voltage and current to produce the optimum power required in each case. Commonly a membrane electrode assembly (MEA) and a bipolar plate (BPP) compose the repeat unit cells. As a place where the electricity can be generated, the MEA is always in the core position of PEMFC. The MEA provides a place for the electrochemical reaction of hydrogen and oxygen, which involves the transmission of mass, heat, and electricity [5]. Therefore, the performance of the MEA definitively and directly affects the output performance and durability of the fuel cell. Shown in Figure 1 is the primary structure of an MEA.

As shown in Figure 1, an MEA can always be classified into three regions: function region (active area), structure region, and transition region. Function region is the core of the MEA, which generate the electric power for the unit cell; structure region provides enough mechanical stability and durability. Although structure region will not improve the electrochemical performance, it plays a role in protecting the proton exchange membrane (PEM), easy handling during manufacturing process, and most importantly for helping to achieve reliable reactant gas and coolant sealing and electrical insulating performance. The transition region is a special region that lies between the active area and the structure region, which is composed of two groups of materials of function region and structure region respectively. The first group, including anode gas diffusion layer (a-GDL), anode catalyst layer (a-CL), PEM, as well as cathode CL (c-CL) and GDL (c-GDL), are called functional materials since they are directly connected to the output performance of the MEA; the second group, including adhesives, resin frames, and seals located on both sides of the PEM, can be classified into structural materials. Traditionally there is a gap between the function region and structure region. For elongated lifetime and better performance, the gap in the transition region can be overlapped, breached, or glued, each kind of structure can be found in the next sections.

Generally, in terms of durability, researchers always pay more attention to CLs, PEM, and GDLs, i.e., all the components within the active area. Usually, vehicular fuel cell operating conditions may cause chemical and physical damage and shorten the life of PEMFCs [6]. Dynamic drive cycle will cause voltage change and accelerate material degradation, which in turn lead to catalyst dissolution and aggregation, carbon carrier corrosion, PEM thinning, and performance degradation [7,8]. Physically, air pressure fluctuations, humidity, and temperature cycling might cause mechanical damage to materials or component structures. Mechanical damage such as pinholes, cracks, tears, etc., may lead to early failure [9]. Changes in temperature and humidity during fuel cell operation can cause the membrane to expand/contract, resulting in in-plane compression/tensile stress. These stresses may be large enough to reach the yield state of the membrane, which may lead to mechanical damage, especially after experiencing hygro-thermal cycles [10,11,12,13,14]. Studies have shown that hygro-thermal stress is the main reason for MEA defects and mechanical performance degradation [15], which has an important impact on the durability of fuel cells [16].

However, the transition region of an MEA is usually ignored, which is of important impact on the lifetime of the whole MEA. The service life of frameless MEA (membrane directly sealing) is usually only a few hundred hours or less. Early failure along the transition region has attracted the attention of researchers [17]. The clamping force, alternated temperature and humidity, and gas pressure fluctuation between cathode and anode during fuel cell operation are the main sources for mechanical damages of this region. Especially, the structural and material differences between active area and structure region are huge, which will produce asynchronous deformation and discontinuity under stress. Improper concept and structural design for this region will incur early failure of MEA. In actual application, the clamping force and the temperature-humidity cycle during operation may cause cracks on the PEM along the frame in a short period of time, resulting in a sudden increase in the amount of gas crossover to an unacceptable level. Therefore, it is difficult to overstate how much the transition region adds to the performance and durability of MEAs, and sufficient attention should be paid to the degradation behavior of the transition region under actual working conditions. Reasonable frame structure design is very helpful to improve the durability of the fuel cell, and is the basis for achieving the 5000 h more vehicular durability goal [18].

This article reviews the literature and patents related the structures, degradation mechanisms and mitigation strategies of MEA, focusing on the transition region. In addition, the article summarizes the durability test protocols MEA such as temperature/pressure shocks appearing in the literature, providing guidance for the rapid validation of transition region design.

It should be pointed out that, all the PEMFCs shown in this review are actually low temperature PEMFC (LT-PEMFCs). Nafion^®^ (DuPont, U.S.A.) membrane of perfluorosulfonic acid (PFSA), whose operating temperature is up to 80 °C, is employed as the electrolyte. For other PEMFCs operating at temperature (HT-PEMFC) higher than 90 °C, Nafion^®^ membrane cannot be used because proton cannot be transferred effectively due to dehydration of the membrane. Phosphoric acid-doped polybenzimidazoles (PBIs) were developed by researchers for HT-PEMFC. However, PBI membrane is quite different from PFSA membrane because liquid H_3_PO_4_ is usually doped into the PBI membrane for adequate proton conductivity. When liquid H_3_PO_4_ appears in the interface between the BPI membrane and the adhesive, a new situation appears if one still want to obtain a reliable and durable structure.

## 2. Structure of Transition Region

### 2.1. The Manufacturing Concept of an MEA

Conventionally, an MEA is fabricated by attaching GDLs to a 3-layer catalyst-coated membrane (CCM), which may be either sprayed or coated via slot die, doctoral blade, or decal transfer processes [19]. Usually, two pieces of discrete GDLs are situated on both sides of the CCM and bonded to it using a hot pressing machine, and then a 5-layer MEA is formed. It should be noted during this procedure, static pressure has been applied to the GDLs and CCM and the thickness of the 5-layer MEA should be fixed according to the design. Frames shown in the structure region in Figure 1 can be made either at the time of 5-layer MEA fabrication, or separately after that.

Frames, which are firmly bonded to electrolyte membrane or other materials in either structure or transition region, provide sufficient robustness and mechanical durability to the MEA. Therefore, although tiny in mass and size, an adhesive with strong adhesion and durability in service environment is critical. Another main function of frames is to achieve robust sealing performance. That is to prevent the reactant gases from leaking into the outside of the fuel cell (external leakage), or crossover between the anode and cathode (internal leakage). As an auxiliary components, the frames and adhesives do not help the electrochemical reaction of the fuel cell. However, the electrochemical reaction cannot proceed normally without the sealing structure.

As for material, considering enough insulativity between the anode and cathode, cheap and easy forming plastic materials with a thickness from 10 μm to more than 100 μm, depending on the thickness of seal members and active area, can be employed, such as polyimide (PI), polypropylene (PP), polyphenylene sulfide (PPS), polyphthalamide (PPA), polyethylene naphthalate (PEN), polyethersulfone (PES), polyvinylidene fluoride (PVDF), polyethylene terephthalate (PET), etc.

Many conventional MEA fabrication methodologies use pressure sensitive adhesives (PSAs). Such PSAs typically have a low glass transition temperature, which can cause the adhesive to ooze or creep during processing or service. Therefore, other rigid crosslinking polymer adhesives have been employed. However, such adhesives generally take an appreciable amount of time to set up relative to their limited pot life. 3M company employed a multi-stage curable resin to bond the GDLs to the CCMs, enabling production of a so-called discrete MEA, with potential high-volume, high-speed, roll-to-roll processes [20].

### 2.2. Sealing Structure of the Transition Region

For a fuel cell stack, reliable and durable sealing of reactant gases and coolant prohibits them from leaking into each other, and the external circumstance is a critical function for MEA designers. Generally, sealing will be reckoned as the function seal members. Sealing materials used in PEMFC are usually elastomers, including polyacrylate, acrylate copolymer, butyl, neoprene, silicone, fluorosilicone, ethylenepropylene-diene-monomer (EPDM), etc., [21]. Novel elastic materials with good resistance to hydrogen, oxygen, water, and heat can also be preferred.

On the other hand, the intact transition region also plays a great important role in maintaining the sealing performance of an MEA. In the short term, if the frame structure is not properly designed, the concentrated stress under clamping force may cause local plastic deformation of the electrolyte membrane, or even tear the membrane directly. In the long run, fatigue and deterioration of materials in the transition region may take place, resulting in sealing structure failure. The frame sealing structure may become a short board that limits the durability of the fuel cell. Although very few papers have been published on the frames and adhesives of MEAs, meanwhile various specific frame structures appear in patents of some FCV manufacturers.

**Figure 2:**
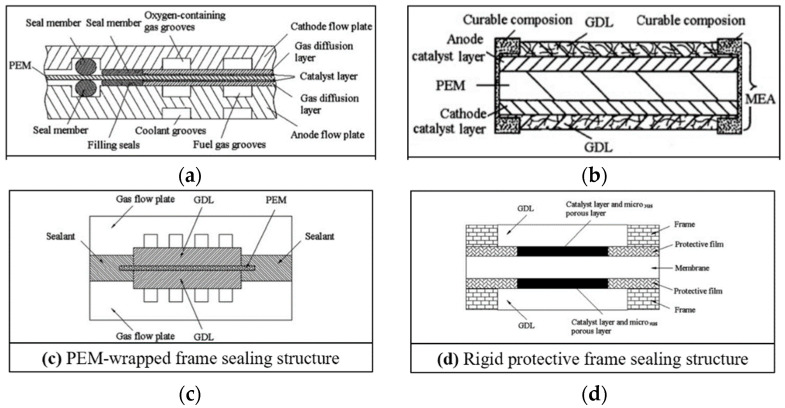
Schematic diagram of the frame structures [22]. (**a**) PEM direct sealing structure; (**b**) MEA-wrapped frame sealing structure; (**c**) PEM-wrapped frame sealing structure; (**d**) rigid protective frame sealing structure.

Previously, Ye et al. [22] have reviewed and classified the sealing structure of fuel cells as four types: (a) PEM direct sealing structure; (b) MEA wrapped frame sealing structure; (c) PEM wrapped frame sealing structure; and (d) rigid protective frame sealing structure. The schematic diagram of the four sealing structures is shown in Figure 2. Among the four types of structures, the membrane direct sealing structure (Figure 2a) is the simplest one, for which the unit cell is sealed by clamping the membrane with two sealing members, without the participation of any adhesive. However, it is difficult to ensure the sealing members on each side of the membrane to be completely aligned. Thus, problems of inadequate sealing, excessive compression of GDL, early failure of PEM, etc., will occur. Although this structure does not have industrial value, it can be used for temporary tests in the laboratory just because of its simpleness. A brief comment needs to be made regarding these four type of structures compared to the simplest structure; adhesive is indispensable in the other three patterns, which in turn bring better applicability. For large-scale production of MEA, the application of adhesive in the structures results in repeatability, robustness, and long-term durability. No matter the adhesive are used for the wrapping/bonding of frame (Figure 2b), PEM (Figure 2c), or double layer frames (Figure 2d). It should be noted that Figure 2 shows only four conceptual forms of frame sealing structure, and various specific implementation cases have been seen in patent and engineering practice since that time.

### 2.3. Integrated MEA Structures

Nowadays compact and reliable MEA structure is favorable in order to achieve the design target of higher specific power density, lower cost, and longer lifetime of a fuel cell stack. Therefore, various components in the transition region are required to be further integrated and polyfunctional. That is to say, the difference between all the components becomes ambiguous. They can be firmly integrated with each other, based upon designers’ overarching goal and innovative concepts.

#### 2.3.1. Seal Integrated onto Separator Plate

**Figure 3:**
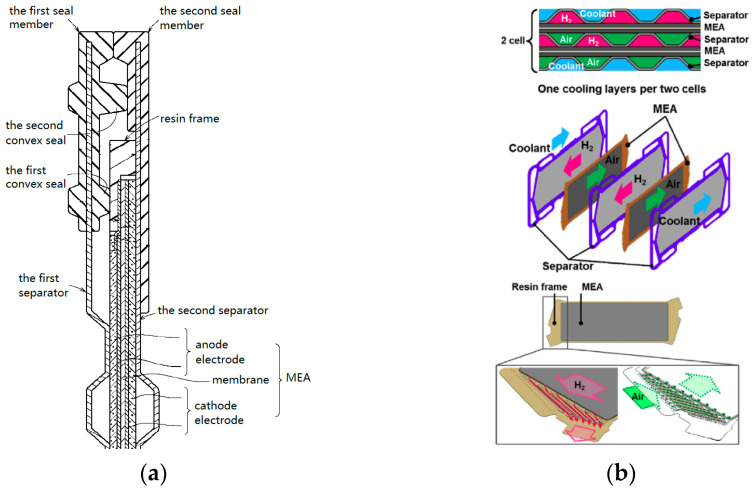
The seal member is integrated on the separator. (**a**) cross-sectional view of MEA [22]; (**b**) fuel cell structure and resin frame [25].

In 2014, researchers from Honda Motor [23] revealed a structure in which seal members were integrated on separator plates, instead of MEAs, as shown in Figure 3a. “MEA-wrapped frame” was adopted, i.e., a resin frame member is joined to the MEA at the transition and structure regions. The first and second seal members were cured on surfaces of both sides of the first separator and second separator, respectively. It look like the separators were “inserted” into the seal members. With this simple and economic configuration, potential damage to the electrolyte membrane can be prevented. In order to further reduce the size of the fuel cell stack, Honda used a waveform configuration which can improve the gas transfer performance of the gas channel and a cooling configuration that represents one unit (two cells) consisting of two MEAs and three separators [24,25]. In addition, with the help of a channeled resin frames positioned in the transition region of MEAs, optimized distribution of H_2_, air, and coolant from the manifold ports to active area has been achieved (Figure 3b).

#### 2.3.2. Frame Bonded with Separator Plate

With the help of adhesive, the MEA frame can be directly bonded with separators to form an assembly, i.e., so-called unit cell in a series patents of Toyota Motor [26]. The adhesive used in the unit cell can be cured with hot pressing, ultraviolet (UV)-curable adhesive, or ultrasonic welding procedures. The process time of a unit cell can be reduced from minutes to seconds by the application of UV-curing process [27].

**Figure 4:**
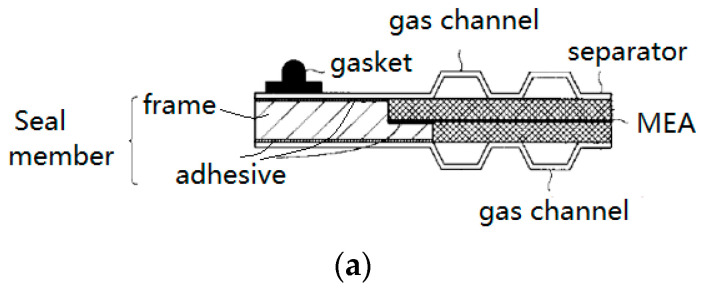

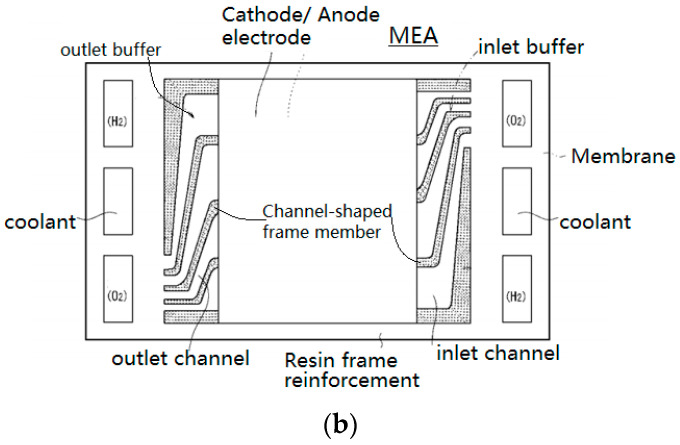
Schematic diagrams of the frame directly bonded to the separator. (**a**) frame as a sealing member [28]; (**b**) channel-shaped members formed on the resin frame [29].

Shown in Figure 4a is an example of this kind of structure. The resin frame of the MEA with a stepped cross section of the inner ring was bonded to the MEA to prevent the crossover of reactant gases [28]. As an intermediate form [29], the flat sides of a frame were respectively bonded to the anode and cathode separators, and a protrusion gasket was molded onto one of the separators to function as the sealing member for the coolant. In this way, the MEA and the separator plates were connected as a whole and could not be disassembled. The frame components can be made of hard materials with good dimensional stability. Instead of forming channels on a separator, it was proposed to make gas guide channels on the frame to introduce gases from the intake manifolds into the flow fields of active area or vice versa from the flow fields to the outlet manifolds [30,31]. Additionally, it can simplify the manufacturing of the separator. As shown in Figure 4b [32], there were reinforced portions on the frame, acting as a buffer channel for reactant gas, thereby ensuring the height of the gas channels and reducing the pressure loss in the gas channels without increasing the thickness of the fuel cell. On the other hand, the reinforcing parts could also be used as a supporting member to prevent the deformation of sub-gaskets [33]. However, if such a structure is adopted, broad buffer channel should be avoided since it might arouse a new problem that the deformation of the GDL or the frame itself will hinder the flow of fluids by intruding into the channels.

#### 2.3.3. Seal Integrated with MEA

**Figure 5:**
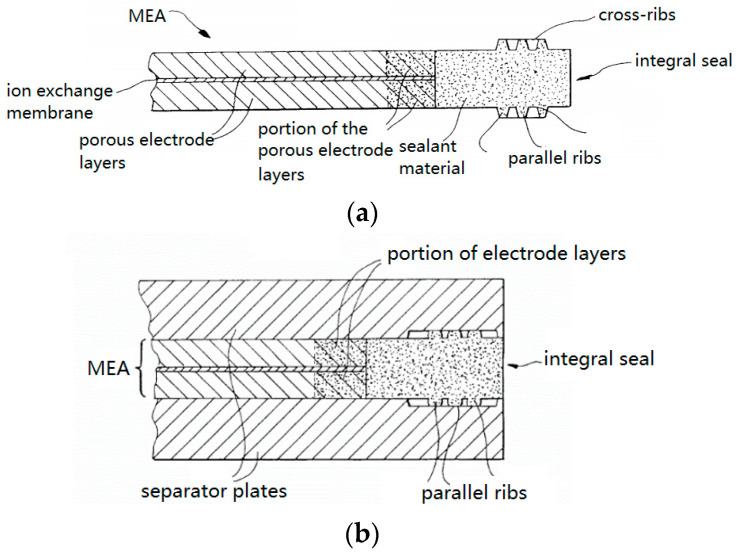

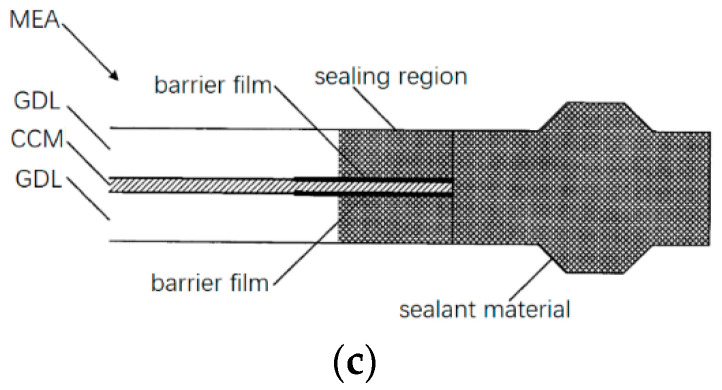
A partial cross-sectional view of the edge of the MEA, impregnate MEA with sealing material to form an integral seal. (**a**) Impregnate the MEA with sealing material [39]; (**b**) two fuel cell separators clamp the integral seal [39]; (**c**) insert a barrier film between the MEA and the sealing material [40].

Another integration pattern for a sealing member is to impregnate the outer circumference of the MEA with a sealing material and extend outward beyond the MEA as an integrated frame [34,35,36,37,38]. These are similar to the MEA-wrapped sealing structure mentioned above. As shown in Figure 5a, a sealing material was impregnated around the end of an MEA and penetrated the pores of the GDLs. Finally, the MEA material was embedded in the sealing material. The sealing material was expected to be an elastomer, which can be molded with a die before curing. One or more convex ribs were formed beyond the structural part of this integrated MEA, whose main function is reactant gas sealing, meanwhile helping to position itself to the separator plates [39] (Figure 5b). In this design concept, sealing material is impregnated into the MEA, therefore the chemical compatibility of the material should be verified in advance. For elastomer materials, permanent deformation under compression force after long-term service is a potential problem and needs to be considered during material screening. In order to avoid possible contamination of the MEA caused by the decomposition of sealing materials, barrier films can be inserted between the membrane and the sealing material [40] (Figure 5c). Of course, extra adhesive is necessary if such a barrier film is used.

The design of the function region structure serves its function of sealing, insulating, and protecting the function region. From the laminated structure of the gas diffusion layer/catalyst layer/PEM in the active area to the frame at the edge, the structure and material have changed. Thus, degradation mechanism in the transition region during long-term operation must be different from the active area, for which numerous studies have been made. Fortunately, researchers have begun to investigate the degradation in the transition region.

## 3. Degradation in the Transition Region

Degradation of the electrolyte membrane, especially the commonly used PFSA, is one of the primary factors of PEMFC performance decay and it can be induced by mechanical stress and chemical aggression, during repeated drying and swelling operating conditions [41].

PFSA was developed in the late 1960s by Dr. Walther Grot from DuPont and registered as Nafion^®^, which was originally suggested to be employed as a selective separator in chloralkali electrolyers [42]. This ionomer consists of the polytetrafluoroethylene (PTFE) main side, perfluoroethylene side chain, and sulfonic group. Although the Nafion^®^ molecules possess stable PTFE backbones, the durability of the PFSA membranes is still not good enough, especially for vehicular application. It has been reported that the lifetime of PFSA under continuous fuel cell operation is in the range of a few thousand to several tens of thousands hours, but depending on operating conditions, such as start/stop cycling, temperature, and relative humidity (hydro-thermal) cycling, etc., [43].

**Figure 6:**
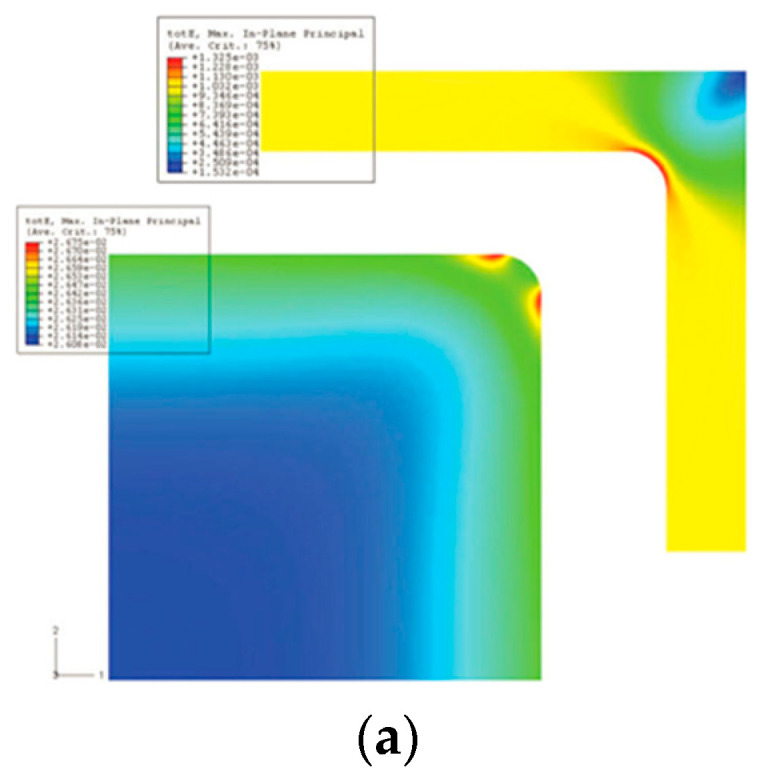

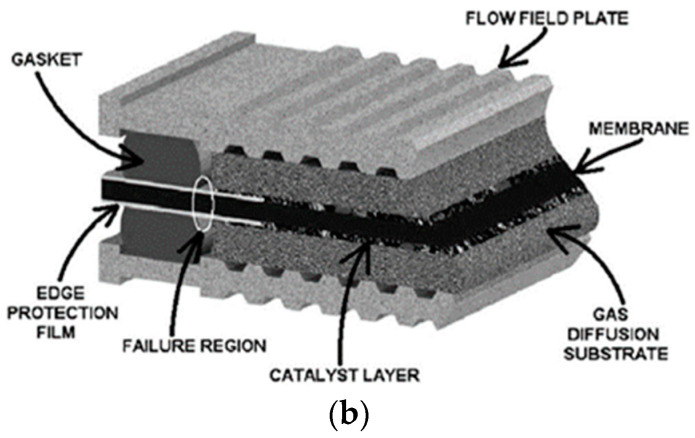
Mechanical damage may occur at the edge of the membrane electrode, leading to early failure. (**a**) Model-predicted distribution of maximum principal tensile strain in the MEA as a result of RH variation from 75 to 0% [47]. (**b**) Schematic diagram of the fuel cell structure, including the location of the protective membrane [49].

Compared with the active area, the degradation in the transition region, especially along the frame is more severe and requires special attention. Li et al. [44] used open circuit voltage test (OCV), low humidity (30% relative humidity (RH)), and high temperature (90 °C) as accelerated stressing test (AST), to perform accelerated degradation experiments of MEA materials. As a result, the MEA performance rapidly decayed after only 48 h, and completely failed after 72 h aging. Their subsequent inspection of the MEA showed that the membrane ruptured at the edge of the catalyst layer under the effect of “edge stress”. Wu et al. [45] studied the MEA durability of a Nafion^®^/expanded polytetrafluoroethylene (e-PTFE) composite membrane using an AST protocol combining RH cycling and load cycling. The results showed that the edge of the membrane showed obvious crack after only 300 h without edge protection. They pointed out that the edge area of an MEA should be protected. As a comparison, under the same AST protocol, the lifetime of an MEA with edge protection and hot-pressing process exceeded 1000 h. Crum et al. [46] studied the mechanical degradation mainly caused by RH cycling in an ex situ inert nitrogen atmosphere. The results showed that the membrane might suffer repeated expansion and contraction due to large changes in local relative humidity. Huang et al. [47] used the finite element (FE) method to simulate and analyze the stress and strain behavior of MEAs under accelerated chemical aging and relative humidity (RH) cycling. The results showed that the process of water uptake and dehydration in membrane stretched and compressed the MEA seriously. The MEA edge area was prone to stress concentration. As shown in Figure 6a, the strain caused by the change in humidity was higher at the edge than in the central area, and the maximum strain occurred near the corner of the frame. In addition, their result indicated that the membrane’s ductility decreased after chemical degradation and RH cycling. The chemical degradation mechanism can be explicated through the unzipping reaction, as shown in Equations (1)–(5).

Early failure at the frame may be caused by the following reasons: (1) Different material characteristics, volume changes due to water absorption and thermal expansion cause concentrated stress under periodic physical loads; (2) membrane edges in particular, gas impact will occur in the area near the air inlet during start/stop; (3) during the MEA manufacturing process, the frame area is more likely to have defects, and there may be bumps and burrs on the frame; (4) hot spots or H_2_O_2_ around the active area may exacerbate the degradation of the transition region of the MEA [44].

MEAs are more likely to be damaged along the frame, and must be properly protected to avoid catastrophic failure. Early failures can be avoided by adding gaskets or applying protective layers [47,48]. The OCV and humidity cycling AST [49] suggested that the lifetime of MEAs with and without the protection of frames were about 300 h and 2500 h, respectively. Electron probe microanalysis (EPMA) results indicated that the membrane without frame protection cracked at the transition region, and the gas crossover suddenly increased to an unacceptable level; meanwhile the gas crossover of the MEA with frame protection increased slowly. However, crossover of hydrogen and oxygen is inevitable, especially when thinner (typically 10~20 μm) electrolyte membranes are employed nowadays to reduce the ionic resistance [50]. Li et al. [44] covered the edges of the membrane by adding a thin adhesive protective layer to the border area. Crum et al. [46] used a set of rigid sub-gaskets to reduce edge effects. The diamond-shaped effective area design employed ribs and channels of the bipolar plate (BPP) at the edges to appear alternately, thereby avoiding local high stress. Using a reinforced membrane could improve the mechanical durability of the membrane. Compared with the non-reinforced membranes, the enhanced membranes have better dimensional stability during hydration/dehydration and strong tear resistance [49]. Under automotive operating conditions, the mechanical stress caused by hydration/dehydration of the reinforced membrane is less and therefore more durable. In the edge area, as shown in Figure 6b, bonding a low-compression frame on both sides of the PEM limited it to the proper position on the edge. The membrane no longer directly bore the mechanical stress from the gasket. In addition, the frame prevented GDL’s stray fibers from puncturing the membrane. In summary, mechanical and chemical damage will occur at the edge of the MEA, leading to early failure of the MEA. Applying a frame protection structure helps to improve the durability of the MEA.

### 3.1. Mechanical Degradation in the Transition Region

Compared to chemical degradation, mechanical degradation is considered to be the main cause of early membrane failure during fuel cell operation. When the fuel cell is exposed to cycling humidity and temperature, the state of stress on the membrane also changes. After long-term service, the mechanical properties of the material gradually deteriorate. When the humidity increases, the membrane constrained by the clamping force tends to be compressed in the plane due to swelling, meanwhile the membrane shrinks and is tensioned at low humidity. Alternating compressive/tensile stress causes degradation and eventual destruction of the membrane. Inherent defects in the membrane or improper stack installation can exacerbate membrane degradation [51]. The temperature gradient inside the fuel cell can cause uneven stress distribution. This may cause local bending stresses, which can lead to delamination between the PEM and the GDL. Uneven stress may be the cause of cracks and pinholes during fuel cell operation, especially under high load conditions [52].

At present, the commonly used bolt fastening and steel belt bundling fuel cell stack fastening methods may cause uneven clamping force distribution on the end plate, BPP, GDL, and at last to the core of each cell, the PEM. On the one hand, excessive clamping force will cause large shear stress at the frame and damage the PEM or on the contrary, cause leakage due to insufficient sealing pressure. On the other hand, current gasket sealing methods tend to distribute a large clamping force at the edges. Bates et al. [53] established a three-dimensional stack model to study the relationship between clamping force and contact pressure and contact resistance. The simulation results showed that the stress in the active area of the laminated MEA was low, and the pressing force was mainly borne by the gasket in the structural area. Improper installation force could cause fuel cell leaks or breakage of fragile internal parts, like graphite BPP. Alizadeh et al. [54] studied the contact pressure distribution through a FE model and verified it with pressure-sensitive films. The results showed that the maximum stress was at the structure area of the MEA, and the contact pressure at the seal was abrupt. The hardness of the seal directly affected the contact pressure at the transition region. The harder the seal was, the more likely the stress concentration occurred. In addition, the assembly error of the BPP and the misalignment of the gasket would also affect the pressure distribution on the active area [55,56]. This might at last result in mechanical damage along the frame and failure of the sealing structure.

As set forth, research on degradation and durability of MEAs mainly focused on the active area. Like in a two-dimensional model of the single channel in the active area [57], when focusing on a single gas flow channel, the stress concentration at the frame has been overlooked [58]. However overall simulation results of the stack [59] showed that even though the stress in the active area was less than the yield stress, the one in the transition area was close to the critical value. Compared to the active area, the transition region is prone to mechanical damage.

A traditional MEA manufacturing process tends to create an overlap or gap between the function area and the structure region [60]. Considering the manufacturing tolerances, components in both regions cannot always be perfectly positioned. In addition, the height difference between the structure region and the active area cannot be ignored during the sealing structure selection and seal member design. Finally, if the GDL covers the frame, overlap occurs in the stacking direction of the fuel cells. All the design bugs may lead to increased stress in the corresponding area on the electrolyte membrane, increasing the risk of mechanical damage. On the other hand, if there is a gap between the frame and the active area, the PEM is directly exposed to cycling temperature and humidity circumstance, leading to irrecoverable problems, e.g., membrane degradation and gas crossover. There have been many studies published on the durability of PEMs under hygro-thermal conditions. Temperature and humidity cycling cause in-plane compressive and tensile stresses, known as hygro-thermal stresses, during which stresses in the PEM may reach the yield limit. Therefore, temperature and humidity cycling are the main causes of PEM and MEA defects. 

Many researchers have used two-dimensional single-channel simulation models to study the effects of hygro-thermal cycles on membranes [13,14,15,57]. The single-channel model is taken from a cross-sectional view of the unit cell perpendicular to the flow channel. This two-dimensional model contains BPPs, GDLs, CLs, and a PEM. Various scenarios can be analyzed by changing the boundary conditions of the model. For example, changing the way the load is applied (displacement, or force applied to the BPPs) to analyze the mechanical response of different stack packaging methods, applying cycling temperature and humidity loads to obtain stress-strain response of internal components during fuel cell operation. These EF simulation with the two-dimensional single-channel model showed that compared with the fixed force, the fixed displacement installation method brings higher stress. In all the cases, the shear stress is small, and the in-plane stress is the largest stress component during the loading process, which controls the yield behavior. Residual tensile stresses may occur during the hydration/dehydration process. These high in-plane residual stresses may be the main cause of mechanical failure of the membranes, and mechanical failure may lead to the generation and propagation of defects. The coupling of the models of computational fluid dynamics and structural mechanics were used to investigate the mechanical response of membranes under real fuel cell operating conditions [61,62]. Instead of a linear uncoupled temperature and humidity profile, the temperature and humidity distribution was derived from the output current of the fuel cell in this model. The results showed that the anode and cathode exhibited different stress responses. The PEM on the cathode side was more likely to be plastically deformed, and the increase in inlet pressure and humidity on the cathode side caused a significant increase in plastic strain. 

Fracture mechanics theory was employed to study the development of cracks on PEM in a hot and humid environment [63,64]. The ex situ uniaxial tensile test under controlled temperature and humidity was performed to simulate the fatigue and mechanical stability of the membrane [65]. Khorasany et al. [66,67] developed a FE fatigue analysis model based on the Smith-Watson-Topper (SWT) method to analyze the fatigue life of PEMs under different temperature and humidity. Theiler et al. [68] introduced the effect of membrane thickness into the life prediction model. Almost all the above research use isotropic linear elastic models with strain hardening plasticity to describe the behavior of the membranes. However, because the mechanical properties of ionomers also exhibit time dependence [69,70,71], linear elastic and plastic models cannot fully respond to continuous or cyclic loads and fully capture the membrane stress and strain. As we all know that the mechanical properties of the membrane are not only related to temperature and humidity, but also to the deformation ratio and time. Therefore, all these factors should be analyzed during the prediction of the fatigue life of a membrane.

**Figure 7:**
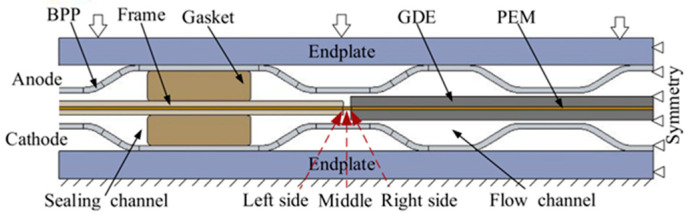
Cross-section view of the FE model of a PEMFC [72].

Except for the active area, the transition and structure regions are exposed to the hygro-thermal environment, subjected to mechanical stressing because of membrane expansion/contraction. Due to the shear stress of the frame and the gas diffusion layer, the membrane on the periphery of the MEA may undergo creep deformation, and the thickness is thinner than other areas. In addition, there are pressure shocks at inlets and outlets of the reactant gases. It has been reported that cracks in the membrane at the transition region cause early failure of the PEMFC [44]. Qiu et al. [72] studied the stress evolution in the membrane between the frame of MEA and GDL during all the processes a fuel cell may suffer such as, assembly, operation, and gas filling. Focusing on the joint-area between the frame and GDL, as shown in Figure 7, a FE model was established, and the component displacement, hygro-thermal conditions, and air pressure were applied in order to determine the mechanical deformation of the membrane. It was observed that the membrane in the frame-GDL joint area experienced severe stress concentration and bending deformation. The pressure difference between the cathode and anode after the introduction of reactant gases caused a rapid increase in in-plane stress, which was the main driving force for the rapid degradation of the membrane. The delamination between the frame and GDL greatly affected the stress concentration. The stress increased with the joint width within a certain range. It was also observed that the concentrated stress was sensitive to air pressure, followed by temperature and relative humidity. Therefore, additional gasket seal or adhesive protective layer are recommended for the bonding of the frame and GDL.

### 3.2. Chemical Degradation at the Transition Region

The chemical degradation of fuel cells is usually caused by free radical attack. As well-known, 2-electron oxygen reduction reaction (ORR) takes place on the cathode platinum catalyst, and hydrogen peroxide (H_2_O_2_) is produced and decomposes to oxidative hydroxyl (HO·) and hydroperoxyl (HOO·) groups. The carboxylic acid groups (–COOH) in the PEM will chemically react with free radical HO to gradually corrode and degrade the molecular chain of the PEM [73]. In the well-known theory of “unzipping mechanism”, trace residual H-containing terminal bonds is the main reason for the generation of -COOH and the decomposition of the membrane [74]. The principle of this chemical reactions is shown in the following reaction Equations (1)–(4).

(1) $$O_{2}+2e+2H^{+}=H_{2}O_{2}$$

(2) $$R-{\mathrm{CF}}_{2}\mathrm{COOH}+\mathrm{HO}\cdot \to R-{\mathrm{CF}}_{2}+{\mathrm{CO}}_{2}+H_{2}O$$

(3) $$R-{\mathrm{CF}}_{2}+\mathrm{HO}\cdot \to R-{\mathrm{CF}}_{2}\mathrm{OH}\to R-\mathrm{COF}+\mathrm{HF}$$

(4) $$R-\mathrm{COF}+H_{2}O\to R-\mathrm{COOH}+\mathrm{HF}$$

The probable decomposition mechanism of the sulfonic acid group is that when the hydrogen atom of the sulfonic acid group is abstracted by the hydroxyl radical, then the $-{\mathrm{CF}}_{2}{\mathrm{SO}}_{3}\cdot$ radical is formed. Next, SO_3_ gas is eliminate and $-{\mathrm{CF}}_{2}\cdot$ radical is formed. Then the unzipping reaction continues (Equation (5)) [75].

(5) $$-{\mathrm{CF}}_{2}{\mathrm{SO}}_{3}H+\mathrm{HO}\cdot \to -{\mathrm{CF}}_{2}{\mathrm{SO}}_{3}\cdot +H_{2}O\to -{\mathrm{CF}}_{2}\cdot +{\mathrm{SO}}_{3}$$

The chemical degradation damages the molecules of the PEM, reducing its integrity, mechanical strength, and protonic conductivity. In addition, it will lead to thinning of the membrane and continuous release of fluoride ions, and will be more prone to pinholes and cracks [76]. The presence of cationic contaminants such as Fe^2+^, Cu^2+^, Al^3+^, etc., which are almost omnipresent in fuel cell environment, may accelerate the membrane degradation [77]. Additionally, the generation process and chemical degradation of free radicals can be accelerated under OCV and low humidity environments [9].

**Figure 8:**
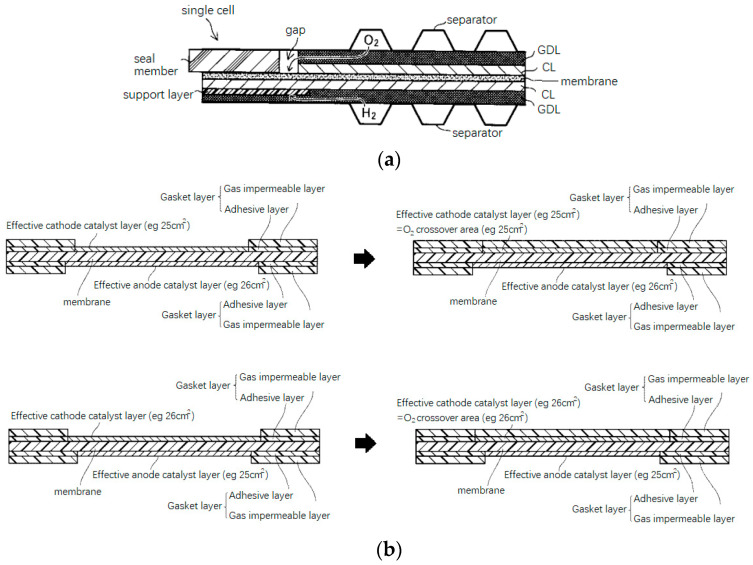
Misalignment of cathode and anode catalysts at the edges of membrane electrodes may aggravate chemical degradation. (**a**) Gap at the joint of the active area and the frame [78]. (**b**) As the area of the cathode catalyst increases, the oxygen passage area becomes larger [79].

As shown in Figure 8a due to design tolerances, manufacturing or assembly errors, there may be a gap at the transition region, leaving the electrolyte membrane directly exposed to the circumstance. Cathode oxygen in the gap can easily contact with protons diffused from the anode and produce H_2_O_2_, (reference to Equation (1)), which will exacerbate the chemical degradation of PEM [78]. On the other hand, when the cathode and anode at the edge of the MEA are not completely aligned (as shown in Figure 8a, the area of the anode catalyst layer is larger than the cathode), the effective catalyst area (the presence of cathode and anode catalysts on both sides of the membrane) will be decided by the smaller area of the anode and cathode. With the same effective catalyst area, if the area of the cathode catalyst layer is larger than that of the anode, the area of oxygen penetration will be larger (Figure 8b) and be beneficial for the production of oxidative free radicals, which is not conducive to inhibiting the degradation of the electrolyte membrane [79]. The patent [78,79] reminds the designers to pay attention to the chemical degradation at the edge of MEA. However, the extent to which chemical degradation will occur at the gap between the MEA active area and the transition region remains to be further studied.

Usually, the edges of an MEA are adjacent to the sealing structure/member. There are even some integrated sealing methods that impregnate the sealing member into the MEA. However, under the working environment of the fuel cell, the sealant may degrade to produce contaminants, which may migrate into the membrane, resulting in membrane contamination and shortened fuel cell life. It has been reported that when silicone is used as a sealing material, siloxanes slowly leach out during fuel cell operation, migrate to the PEM, and then be chemically oxidized to silica derivatives [80]. The accumulation of decomposition products from silicone rubber in the MEA may change its wettability, and the fragments of silicone may result in a poisoning effect on the platinum catalyst [81]. Contamination can be reduced or eliminated by making the sealing area substantially free of active electrocatalyst particles or inserting a barrier film between the electrolyte membrane and the sealant material [40] (Figure 5c).

### 3.3. Measures to Mitigate Transition Region Failure

The transition region consists of materials with varying properties such as metal, graphite, high molecular polymer, and adhesives etc. With the change in operating temperature and humidity, all the materials exhibit different expansion and contraction ratios, leading to shear stress between the components. Besides, considering the error during manufacturing processes, a gap will be left between the middle CCM and the peripheral frame. Wherein the electrolyte membrane, locating in middle of the layered MEA, is directly exposed to water and reactant gases, and the PEM will expand and contract due to water uptake/dehydration and temperature changes, and will degrade due to the corrosion of free radicals. How to avoid the direct exposure of the PEM in the gap to the working environment is the most critical consideration that improves the durability of the MEA. How to design the transition region, making this area free of structural defect and stress concentration are the key points to ensuring material and structural continuity. In order to improve the durability of this area, the researchers proposed various structure designs, which are inevitably related to bonding adhesives, GDLs, frames, and electrolyte membranes.

#### 3.3.1. Bonding Adhesives

As for adhesive, ethylene propylene diene rubber (EPDM), with properties of low price, anti-oxidation, aging resistance, and water-resistance, is used as the seal member by Toyota Motor [82] and Honda Motor [83]. Other than EPDM, polymer elastomers such as nitrile butadiene rubber (NBR), fluororubber, silicone rubber, fluorosilicone rubber, butyl rubber, natural rubber, styrene rubber, chloroprene rubber, or the like can also be used. All the materials can be thermosetting or thermoplastic.

The injection, coating, or dispersion process of viscous adhesives differs from the application of solid state materials. Uneven dispersion, bubbling, or oozing of adhesives will affect the bonding quality and reduce the connection strength [84,85]. High-frequency vibration on the adhesive coating surface can make the adhesive spread evenly and quickly. Therefore, as the most efficient and convenient approach, ultrasonic vibration via the resin frame are usually used to reduce air bubbles in the adhesive. However, it should always be kept in mind that reasonable ultrasonic power and time should be adopted to avoid potential damage to CLs due to vibration.

Usually thermoplastic or thermosetting adhesives are used as adhesives to bond the components in structure and transition regions, thus heat treatment is necessary. A typical issue, i.e., the MEA deforming during the heating process, may be incurred. Therefore, UV curing adhesives were proposed [86,87] and adopted by Toyota Motor in the newest FCV [27], wherein a UV-curable adhesive was applied to bond another thermoplastic adhesive, a electrolyte membrane, a CL, and a GDL. Instead of few minutes needed by thermal process, UV-curing takes only few seconds, and potential troubles caused by over-heating of the active area can be avoided; meanwhile deformation and the warpage of the MEA caused by higher temperature and longer processing time can also be suppressed. Therefore, the production efficiency may be greatly enhanced by employing UV-curing process. However, this approach requires the frame substrate of the UV-curable adhesive to possess good ultraviolet light transmittance. In addition, the thickness of the adhesive layer and the surface curing speed need to be carefully matched to avoid uneven and incomplete curing.

#### 3.3.2. Gas Diffusion Layer

**Figure 9:**
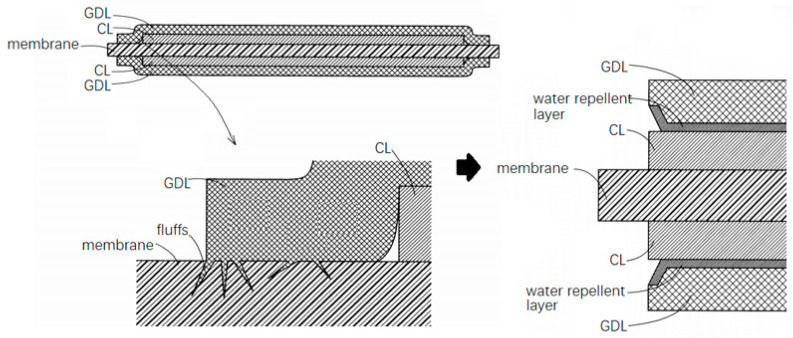
Measures to avoid stray fiber damage [90].

GDL is a conductive and hard carbon fiber-based material. Stray carbon fibers in the substrate, namely so-called carbon paper, may pierce the electrolyte membrane, leading to the reactant gases to pass through [88]. The problem gets prominent at the edges of a GDL, due to the carbon paper cutting process. Mitsuda et al. [89] used elastic rollers to press the raw material carbon paper, and then blown on it to remove the stray fibers; optional means such as electrostatic adsorption and solvent impregnation were also adopted. In another patent [90], it was proposed to incline the contact area between a GDL and a CL by cutting away the sharp edges of the GDL or making a hydrophobic layer. As can be seen in Figure 9, a hydrophobic layer was formed on the surface of the edge-cut GDL, and the area of the catalyst layer was larger than that of the GDL to protect the electrolyte membrane.

#### 3.3.3. Resin Frame

**Figure 10:**
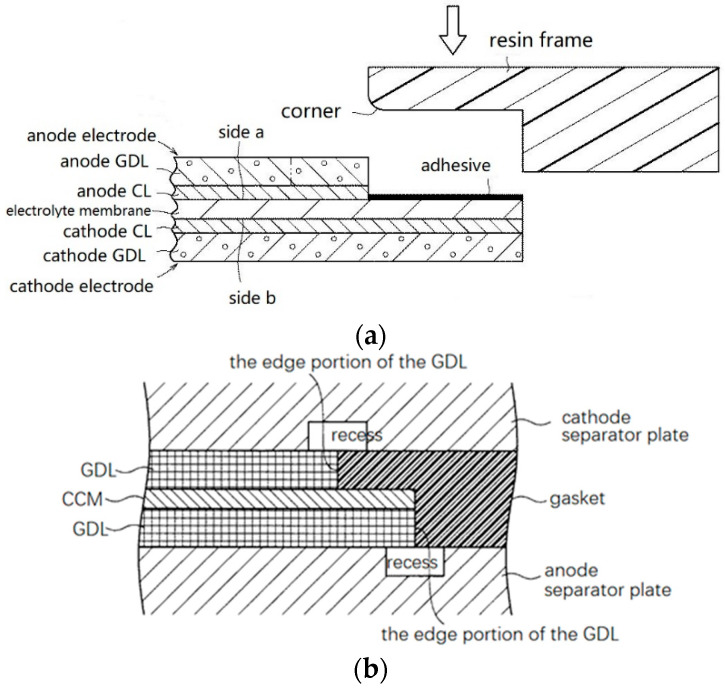
Measures to relieve shear stress. (**a**) stepwise MEA and resin frame [91]; (**b**) recesses formed on the cathode and anode separators [96].

As previously mentioned, resin frames are generally used in both structure and transition regions, and even stretch into the function region. A resin frame is bonded to the CCM and exert its supporting function. However, under the compression of the clamping force, the edge of the rigid frame might be pressed into the electrolyte membrane, thereby generating shear stress on it. When the electrolyte membrane expands, the shear stress becomes more severe. To alleviate the damage of excessive pressure, the corners of the frame where the PEM is adhesive-contacted can be made round [91]. As shown in Figure 10a, a stepwise MEA and resin frame are designed, the corner portion of the resin frame was formed into a curved surface (R shape), which came into contact with the electrolyte membrane. With such a simple configuration, the damage of electrolyte membrane can be suppressed. Naoki et al. [23] proposed to apply an elastic adhesive layer as a buffer layer around the periphery of the resin frame to prevent it from being pressed into the membrane directly. The frame protective film covered the periphery of the electrolyte membrane with one end, and covered the surface of the CL with the other end. The mechanical load on the electrolyte membrane was relieved by the protective film.

On the other hand, in terms of dimension the electrolyte membranes to be bonded is not as stable as the resin frame. Moreover, the swelling and contraction of the membrane with respect to relative humidity were nearly two times greater than for the CCM [92,93]. Additionally, swelling and contraction due to hygro-thermal variations leads to non-uniform expansion that may exacerbate local degradation. Reducing the in-plane and through-plane helps improve the shear resistance of the membrane [94]. Based on mechanics, uniform coating of the catalyst layer at the edge can ease the shear of the frame to the membrane and improve the uniformity of the stress distribution. However, this approach increases the amount of expensive precious metal catalyst, which is adverse to cost control. Instead of homogeneously coated catalyst, the content at the edges of the CL can be lower than that in central area, or other non-conductive and cheap substitute materials can be used as long as they are close in mechanical properties. Another benefit of the employment of non-conductive substitute material is that it can eliminate electron conduction paths and suppress the generation of hydrogen peroxide [95].

To reduce the shear stress applied to the membrane at the edges of the GDLs and the resin frames, recesses were designed in both anode and cathode separators (Figure 10b). These recesses also contributed to suppress the creep deformation and thinning of the electrolyte membrane [96]. However, because of the crossover and retention of reactant gases and water in the recess area, chemical degradation may be triggered according to the mechanism mentioned in Section 3.2. On the other hand, the structure of the BPPs is limited since the edges of an MEA is usually straight, while curved flow field channel design is popular for better reactant gas and water transfer under higher current densities. Therefore, how to match the straight MEAs and curved flow channels should be carefully conceived.

#### 3.3.4. Gap Problem

**Figure 11:**
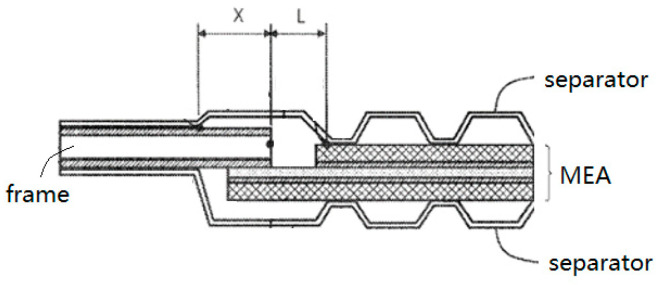
Unit cell structure diagram.

When a fuel cell encounters varying temperature from −30~80 °C, the frame and the CCM expand or contract in opposite directions, which may cause catastrophic rupture of the PEM. Shizuku et al. [97] pointed out that a unit cell structure should be designed in line with Equation (6) to avoid the rupture (Figure 11).

(6) X×ΔT×CTEf<(1−ΔT×CTEm)L×t

**Figure 12:**
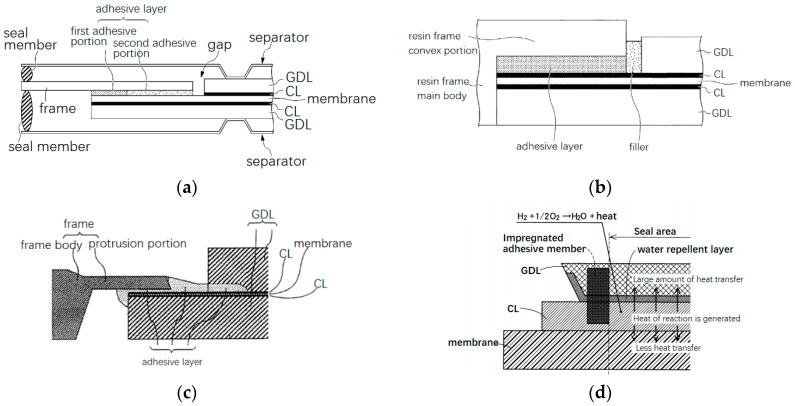
Measures to resist peeling and alleviate degradation in gaps: (**a**) two adhesives are used to bond the frame and MEA [98]; (**b**) filler is applied at the gap [108]; (**c**) adhesive covers gaps and edges of CCM [108]; (**d**) adhesive is impregnated into the edge of MEA [90].

As shown in Figure 11, X indicates the length of the frame that is not constrained by the separator at high temperature, and L indicates the length of the membrane from the end of the frame to the separator; ΔT represents the temperature change range from high temperature to low temperature; CTEf represents the total average coefficient of linear expansion of the resin frame; CTEm represents the linear average expansion coefficient of the electrolyte membrane; t represents the elongation at break of the electrolyte membrane at low temperature. When the temperature changes, the resin frame may peel off from the membrane electrode due to cracking of the adhesive. Tanaka et al. [98] pointed out that when the temperature changed, the frame and the MEA tend to peeled off from each other, and the shear stress near the CCM side was greater than that near the frame side. The concentrated stress can be effectively suppressed by applying a binder with a lower Young’s modulus on the inner side near the CCM (the second adhesive as shown in Figure 12a) and the other binder with higher Young’s modulus on the outer side (the first adhesive in Figure 12a). However, even two kinds of adhesives have been used, the gap is still exist with potential risk. Due to pressure fluctuations, expansion and contraction, and chemical degradation, the electrolyte membrane exposed to the gap between the active area and the frame is easily to be perforated and torn.

Takasaki [99] revealed an approach with purpose to avoid the damage of the gap position by applying surface pressure on the membrane to restrict its position and reduce the effect of expansion and contraction. Another method is to isolate water and gas from getting into the gap and contact directly with the PEM. It can be realized by filling the space with adhesive, or impregnating resin into the end portion of the gas diffusion layer. The idea has been manifested in a patent, wherein the gap between the frame and the membrane electrode was filled with an adhesive filler with an elastic modulus of 1 to 30 MPa at 23 °C (Figure 12b), which can suppress the separation of the structure region and the active area when the temperature changes [83]. In order to increase the bonding strength between the two regions, resin can be impregnated at the outer circumference of the materials in the two regions and combined them together [100,101,102]. In this way, gaps can be really avoided. However, the processing complicacy increases, and the production method of the MEA needs to be optimized, especially for large-volume manufacturing.

There are still numerous innovations that have been conceived to makeup the gap issue. Kimura [103] covered the membrane at the end of the MEA with a gasket to avoid damage caused by pressure fluctuations and pressure difference between anode and cathode sides. Saito et al. [104] disposed water-absorbing member, e.g., a highly water-absorbing cellulose material or an acrylic acid polymer partially sodium salt cross-linked product in the gap. The water-absorbing member was partially compressed by the separator and partially exposed to the flow channels. After absorbing water, the component expanded to produce downward surface pressure, thereby suppressing the expansion deformation of the PEM and avoiding the accumulation of mechanical stress. Kusakari [105] believed that the bending stress generated by a pretilt angle in the periphery of an MEA could help suppress deformation. Through the design of the unit cell structure, the end of the MEA was inclined downward in advance, and covered with adhesive to generate pre-stress to resist deformation. By partially covering the CCM exposed on the cathode side with an adhesive, water and gas could be isolated and fatigue due to expansion and contraction was suppressed [106]. If the edges of the CCM cannot be covered, there was a high probability that it will crack in a long run due to repeated expansion and contraction [107]. Kawasumi [108] pointed out that the peripheral part of the GDL should be placed inside the adhesive and connected to the CCM through a thin layer of adhesive (Figure 12c). It is necessary to cover the gas diffusion layer before the adhesive is cured, so that the adhesive can penetrate into the pores of the porous medium, so as to prevent the cured adhesive from cushioning the GDL and generating stress concentration.

The impregnation of resin or adhesive member in the outer periphery of the GDL can suppress the diffusion of gas into the gap and prevent the membrane from being deteriorated due to hydrogen peroxide attack. The heat generated by the reaction in the active area can be transferred through the GDL with high thermal conductivity. The resin frame member at the edge has low thermal conductivity, and the heat cannot be dissipated in time, which tends to aggravate the expansion of the electrolyte membrane (Figure 12d). The area of the CL is larger than the area of the GDL, which can also act as a protective barrier to prevent stray fibers from puncturing the electrolyte membrane. Oxidative free radicals in the catalyst layer is more easily decomposed, which helps to inhibit the chemical degradation of the electrolyte membrane [90,109].

This section summarizes some typical measures and methods in both designing and manufacturing processes of the transition region MEAs, most of which are reported in patents. Bonding the region with UV-curing process can avoid the warpage caused by the asynchronous deformation of the components. The gaps between the structure region and active area must always be avoided, by applying adhesives, fillers etc. Impregnating adhesive into the transition region can also improve the bonding strength and peel resistance, thus MEAs with higher robustness and durability can then be achieved. Others measures such as laying a protective film and setting recesses on the separators can help alleviate the shearing effect of the rigid frame on the PEM.

As a structural material of an MEA, it should always be kept in mind that the resin frame is not independent of the whole of the sealing structure, and its matching with other materials in the transition region should be considered when designing the MEA.

## 4. Durability Test Methodology for Transition Region

As previously stated, the structure of a transition region of an MEA is important to the durability of PEMFC, hence more and more adhesive materials are used. As a special structural material in this region, the durability of frame, as well as adhesive layer on it, must be evaluated in an efficient way before real application. Actually, a frame is a 2-layer or 3-layer structure, composed of substrate layer and adhesive layer(s). Usually, the substrate layer must be mechanically and chemically stable and reliable. Compared to the substrate layer, the selection of suitable adhesives is more challenging since it directly contacts with the PEM, for which PFSA is exclusively used and the PFSA membrane is difficult to be bound. Furthermore, ubiquitous water in the MEA, no matter in gas or liquid state, is easy to permeate into most of the adhesives or the interfaces between the adhesives and other materials, e.g., the frame substrate layer, PEM, GDL, etc. Then bonding strength might be weakened, followed by catastrophic malfunction or failure of sealing.

The mechanical degradation of the transition region can be tested by means of ex-situ tensile test and in situ test under cycling temperature and/or humidity. No matter what kind of test method is adopted, the robustness and endurance of an adhesive becomes the crucial purpose for the transition region durability test. Sealing performance validation test should focus on the reliability of the adhesive. It can also be found in literature, which usually involve connection strength between the CCM and the frame, anti-stripping ability of frame, and the mechanical degradation test etc.

Usually in situ fuel cell test is relatively easy to implement with either real unit fuel cell or stack. Postmortem characterization and analysis of the materials in transition region after accelerated degradation test (duty cycle, OCV, etc.,) can also be employed to investigate the degradation mechanism in the in situ fuel cell environment. Kawasumi [108] et al. measured the bonding strength between the membrane electrode and the support frame through a tensile shear bond strength test. The mechanical properties of the frame at different temperatures and humidity can be characterized by the tensile testing. Mitsuda et al. [83] designed a resin frame peeling resistance evaluation protocol, i.e., thermal shock. Nitrogen gas with different temperatures were alternatively introduced into both the anode and cathode gas channels of a fuel cell stack to test the bonding strength between the frame and the PEM. The periodical exposure temperature and time were controlled at 70 °C 5 min and −20 °C for 5 min to mimic the freeze-thaw cycle, and a real stack may be challenged. The protocol was repeated thousands of times before the amount of gas crossover between the anode and cathode being measured to confirm whether the frame was peeled off from the membrane electrode. Another patent [98] reported a similar method, in which N_2_ with high and low temperature was alternatively introduced into the cathode and anode flow channels, acting as the internal environmental stressing medium. Except for the exacerbated high temperature (95 °C), which is higher than current stack operation temperature, all the other processes have been succeeded from reference [83].

Similar to thermal shock test, pressure shock test can also be carried out in a simpler way. In 2007, Garland [110] proposed an MEA durability testing protocol, in which the gas pressure of a PEMFC was alternated between 10 kPa and 200 kPa for every 15 min. Gas crossover between anode and cathode was also employed as the indicator to judge if the adhesive is peeled off from the PEM. The cycling test in a nitrogen environment isolates the mechanical durability associated with membrane expansion and contraction. However, if air and hydrogen are supplied to a unit cell, both the mechanical and chemical degradation are involved.

According to RH cycling test proposed by the United States Department of Energy (DOE), the cathode and anode of a unit cell are ventilated with air, and the internal humidity of the fuel cell is periodically changed. The durability of an MEA can be indicated by the number of test cycles observed before the gas crossover reaches the threshold [110]. The specific test protocol of DOE for mechanical durability of MEA is shown in Table 1 [111].

More durability testing methods can also be retrieved in the literature [111]. For example, there are many accelerated test methods for the function region of an MEA, such as OCV and start-stop cycle testing, include mechanical and chemical degradation. Characterization methods such as bubble test and infrared thermal imaging have been reported to detect the exact location of gas crossover [112,113]. SEM, XCT, etc., were used to observe the morphology of the samples after accelerated testing [59,64]. Certainly, besides the degradation of the function region, the degradation of the transition region occurs simultaneously. Therefore, the test methodology for function region can also be applicable to the transition region.

It should be pointed out that although rarely reported, ex situ tests on the frame substrate and adhesive are more helpful for understanding the aging mechanism of the transition region in environment with certain temperature, humidity, acid, and compression force, etc. Of course, special experimental tools, equipment, and method must be developed other than in situ test.

## 5. Conclusions

Significant advances in the past decades have brought MEAs to a stage where they are becoming industrial product. Unfortunately, state-of-the-art MEAs are widely observed to be suffering from insufficient reliability and stability. This review summarizes the literature, especially patents, on MEA transition region, focusing on the structural patterns, degradation, and durability test methods. The following conclusions can be drawn:

1. Aiming at higher power density, an MEA with robust and compact structure, especially with an integrated transition region, is preferred. Bonding the materials in the transition region with adhesives, even integrated with separators, can reduce the number of components and simplify the unit cell structure. The integrated sealing structure reduces or eliminates the stress concentration on the electrolyte membrane and helps to improve its durability. However, considering its non-amenable nature and high requirements of the accurate positioning and quick processing of the integrated fuel cell, automated processing technology is necessary. However, except for a limited number of plants, which possess not only a big demand for capacity but also a mature manufacturing process, hand or semi-automatic manufacturing is still more practical for most of the stack makers.
2. The durability of MEAs has received widespread attention, but there are relatively fewer research on the durability of transition region. At present, when MEAs are produced, there will be gaps or overlaps between the active area and the structure region. The overlapping of the components increases the risk of stress concentration. PEMs exposed to the gaps are susceptible to mechanical damage and chemical degradation. Under the influence of compression force, temperature, RH, and gas pressure, the deformation of the materials at the transition region is not synchronized and tends to peel off from each other. In order to improve the durability of this region, researchers proposed many strategies, among which avoiding exposure of PEM to water and reactant gases, limiting the area of this region is the main principle.
3. To validate the endurance of the transition region, in situ thermal shock, pressure shock and dry-wet cycles were used to test the mechanical durability; to couple both mechanical and chemical degradations, open circuit voltage, RH cycling, and temperature cycling under reactant gases were employed simultaneously. The amount of gas crossover is usually used as an indicator to assess the severity of such degradation. In future, for further development of transition region to meet the durability requirements of automotive fuel cells, novel designs as well as ex situ test methods are necessary. It is also necessary to further evaluate it under dynamic driving cycle, lower temperature such as −30 °C or less, etc.

## Figures and Tables

**Table 1:** DOE AST protocol for MEA mechanical degradation [111].

Cell operating conditions	Unit cell 25–50 cm^2^ (test using an MEA)	
	Temperature: 80 °C	
	Fuel/oxidant: air/air at 2 slpm on both sides	
	Pressure: ambient or no back-pressure	
	Step change: cycle 0% RH (2 min) to 90 °C dewpoint (2 min)	
Cycle	Cycle time: 24 h	
	Total time: until crossover >10 sccm or 20,000 cycles	
Metrics	Frequency	Target
Crossover	Every 24 h	≤10 sccm
